# Risk factors for low back pain after oblique lumbar interbody fusion in patients with low-grade degenerative lumbar spondylolisthesis: a retrospective study

**DOI:** 10.3389/fsurg.2024.1494849

**Published:** 2025-01-06

**Authors:** Shuanchi Wang, Jiabao Chen, Zhe Lu

**Affiliations:** ^1^ Department of Orthopaedics, Cangzhou Hospital of Integrated Traditional Chinese and Western Medicine, Cangzhou, Hebei, China; ^2^ Third Hospital of Hebei Medical University, Shijiazhuang, China

**Keywords:** low back pain, OLIF, risk factors, mild degenerative lumbar spondylolisthesis, prognosis

## Abstract

**Objective:**

To investigate the risk factors of low back pain after oblique lumbar interbody fusion (OLIF) in patients with low grade degenerative lumbar spondylolisthesis (DLS).

**Methods:**

This retrospective study included 116 patients with single-level low-grade lumbar spondylolisthesis with low back pain who underwent OLIF surgery in our hospital from December 2017 to October 2020. Demographic, clinical, surgical, and radiological characteristics of this population were analyzed to determine the relationship between these characteristics and the degree of low back pain relief after OLIF.

**Results:**

A total of 116 patients (average age 58.61 ± 7.01 years) were included in this study. Among them, 33 patients had poor improvement of low back pain after surgery, and 83 patients had satisfactory effect after surgery with obvious relief of low back pain. Postoperative disc height increase ≤2.5 mm (*P* = 0.000) was most correlated with poor improvement of low back pain symptoms after OLIF. The factors positively correlated with poor improvement of low back pain symptoms after OLIF in patients with low-grade degenerative lumbar spondylolisthesis included the increase of spondylolisthesis grade (OR = 17.665; 95%CI: 3.262–95.678 *P* = 0.001), disc height increase ≤2.5 mm (OR = 34.377; 95%CI: 5.632–209.818 *P* = 0.000). The factors negatively correlated with poor improvement of low back pain symptoms after OLIF in patients with low-grade degenerative lumbar spondylolisthesis included no osteoporosis (OR = 0.067; 95%CI: 0.013–0.350 *P* = 0.001), no cage subsidence (OR = 0.208; 95%CI: 0.048–0.903 *P* = 0.036), duration of preoperative low back pain symptoms ≤36 months (OR = 0.045; 95%CI: 0.007–0.277 *P* = 0.001).

**Conclusions:**

OLIF can significantly improve the low back pain symptoms in patients with low-grade degenerative lumbar spondylolisthesis. High grade of spondylolisthesis before operation, duration of low back pain symptoms more than 36 months, osteoporosis, postoperative cage subsidence, and postoperative segmental disc height improvement less than 2.5 mm are risk factors for low back pain after operation.

## Introduction

Lumbar spondylolisthesis is a common spinal disease. According to the Wiltse classification system, there are five types: degenerative, dysplastic, traumatic, isthmic lesion and pathological fracture lumbar spondylolisthesis, among which degenerative lesion is one of the most common types (1–3). The clinical manifestations of degenerative lumbar spondylolisthesis are usually different, usually including low back pain, radiating pain and numbness of the lower limbs, intermittent claudication, and even lower limb weakness, numbness in the saddle area, bowel and urinary dysfunction caused by traction or compression of the cauda equina nerve. Lumbar pain is the most common symptom of degenerative lumbar spondylolisthesis. These symptoms are usually related to biomechanical instability of the spondylolisthesis site. Spinal instability leads to disc degeneration and lumbar spinal stenosis, which eventually leads to invasion of nerve roots and dural sac. Adult patients with degenerative spondylolisthesis with severe symptoms, serious impact on patients’ daily life, long duration, and ineffective conservative treatment are usually treated by surgery (4, 5). At present, various surgical approaches mostly treat low-grade degenerative lumbar spondylolisthesis by achieving spinal stability, nerve decompression, intervertebral disc space height restoration, and deformity correction (6, 7). In order to reduce the complications associated with traditional open surgery, surgeons have developed minimally invasive techniques such as oblique lumbar interbody fusion (OLIF).

OLIF refers to direct access to the responsible intervertebral disc through the retroperitoneal abdominal vascular sheath and the physiological space of the anterior edge of the psoas major muscle, and after resection, the fusion cage is inserted to increase the height of the intervertebral disc, reduce the prolapse of the intervertebral disc, and prolong the hypertrophic ligamentum flavum to achieve indirect decompression and interbody fusion. In OLIF, the large cage is placed from the lateral side of the vertebral body, and the bone graft area is large and the intervertebral space height is restored. OLIF can not only reduce the destruction of bone structure, but also reduce the exposure of spinal canal, avoid the traction of nerve root, reduce the occurrence of cerebrospinal fluid leakage, nerve edema and other related complications, and the long-term fusion rate is also good (8–12). Compared with traditional surgery, OLIF also has the advantages of avoiding the destruction of paraspinal muscles, less bleeding, less trauma, and shorter operation time (13).

Low back pain is often the most common symptom of degenerative lumbar spondylolisthesis. The symptoms of low back pain are usually related to biomechanical instability of the spondylolisthesis site, intervertebral disc degeneration, damage of the peripheral nerves of the articular process, and aseptic inflammation of the lumbar muscles. The Roland-Morris Disability Questionnaire (RMDQ) consists of 24 questions related to low back pain. To distinguish between other causes of dysfunction, each question was restricted by “due to low back pain,” making it easy for patients with low back pain to select. The RMDQ was used to evaluate the status of patients with low back pain during the 24 h before the test. The problems included 8 aspects: walking, standing, bending down, lying in bed, dressing, sleeping, self-care, and daily activities. Chiarotto A scholar in New Zealand searched 6 commonly used international databases to evaluate the validity of disability in patients with low back pain. The results showed that RMDQ had moderate to excellent validity, and the construct validity was better than ODI. The RMDQ is simple, easy to operate, does not require training for assessor and assessor, and is more sensitive for evaluating patients with mild and moderate dysfunction. Since this study mainly focuses on the risk factors of postoperative low back pain in patients with mild degenerative lumbar spondylolisthesis, RMDQ can better evaluate patients’ low back pain (14, 15).

OLIF surgery can relieve the neurological symptoms of lower limbs through indirect decompression for lumbar degenerative diseases, while the relief of low-grade degenerative lumbar spondylolisthesis accompanied by obvious low back pain is usually considered to be related to the stability of responsible segments (disc height recovery and effective fusion of intervertebral space) and the recovery of lumbar curvature (16–18). However, in clinical cases, many factors such as the severity of spondylolisthesis, osteoporosis, body weight and combined internal fixation devices can affect the recovery of symptoms after OLIF. The purpose of this study is to evaluate the effect of OLIF in the treatment of patients with low-grade degenerative lumbar spondylolisthesis mainly with low back pain, and to investigate the risk factors affecting the relief of low back pain symptoms after OLIF in patients with low-grade degenerative lumbar spondylolisthesis mainly with low back pain, so that clinicians can intervene in advance and how to improve the effect of surgery on relieving low back pain symptoms.

## Materials and methods

### Study design and patient population

This retrospective clinical study included patients who underwent OLIF surgery due to low-grade degenerative lumbar spondylolisthesis in our hospital from December 2017 to October 2020. All patients were diagnosed as degenerative lumbar spondylolisthesis by x-ray plain film (19). The inclusion criteria were as follows: (1) diagnosis of degenerative lumbar spondylolisthesis with low back pain; (2) Grade I and II spondylolisthesis according to Meyerding classification (<25% < 50%); (3) conservative treatment for more than 6 months; (4) no history of lumbar surgery; (5) follow-up >36 months. Exclusion criteria were as follows: (1) patients with trauma, reoperation, tumor, infection, congenital deformity, and immune system diseases such as rheumatoid arthritis, ankylosing spondylitis; (2) LBP or radiculopathy associated with extraspinal causes; (3) patients with high degree of sliding (grade III, IV); (4) other types of lumbar spondylolisthesis; (5) patients who underwent secondary surgery at the same or adjacent level.

Thirty-four patients were lost to follow-up due to the visual analogue scale (VAS) and Roland-Morris Disability Questionnaire (RMDQ) before and at least 3 years after surgery in this retrospective study. Eight patients who underwent a second surgery due to infection, poor fusion of the intervertebral space after surgery, acute nerve edema, etc. were excluded from the study. A total of 116 patients (100%) were enrolled in this cohort. The patient's age, gender, body mass index, spondylolisthesis grade, spondylolisthesis segment, preoperative hypertension, diabetes and other basic diseases, duration of symptoms, lumbar lordosis Angle, surgical segment lordosis Angle, intervertebral disc height, and whether percutaneous pedicle screw fixation combined with treatment were recorded before operation, before discharge and 3 years after operation.

All patients underwent lateral lumbar x-ray examination in standing position. The preoperative and postoperative lumbar lordosis Angle, segmental lumbar lordosis Angle and intervertebral disc height were measured by four attending doctors with more than 5 years of experience in spine surgery.

Cage subsidence and bone fusion were assessed using three-dimensional thin-slice computed tomography (CT) images and axial, coronal, and sagittal reconstructions obtained at 36 months after surgery. An interbody fusion cage was considered to be present if it sank >2 mm into the adjacent vertebral body. Bony fusion was defined using the fusion grading system of Bridwell et al. (20, 21).

### Clinical and radiological outcome measures

Lumbar lordosis Angle (LL): The lumbar lordosis Angle between the upper endplate of the L1 vertebral body and the upper endplate of the S1 vertebral body was measured, repeated three times and averaged.

Segmental lumbar lordosis Angle (SL): The Angle between the upper endplate line of the upper vertebral body and the lower endplate line of the lower vertebral body at the surgical level, repeated three times and averaged (22, 23).

**Figure 1:**
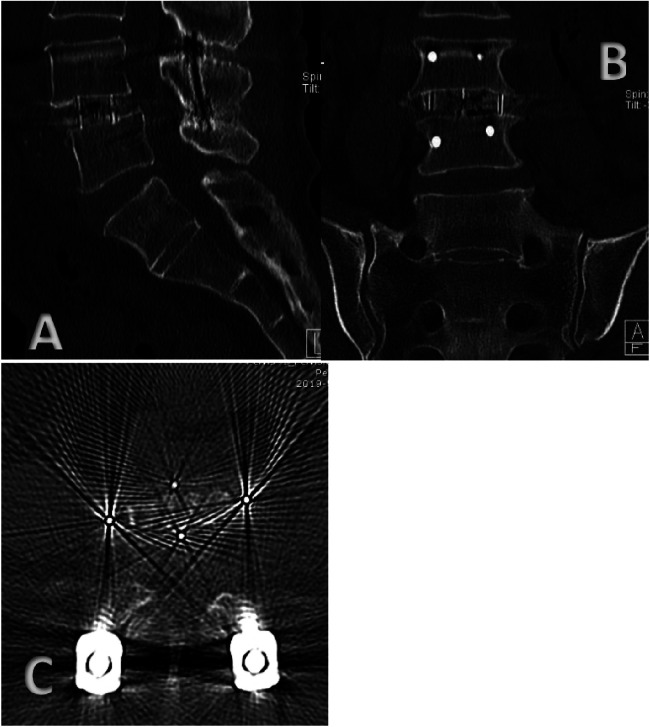
**(A)** Sagittal CT imaging three years after OLIF combined with percutaneous pedicle screw fixation (PPSF). **(B)** Coronal CT imaging three years after OLIF combined with percutaneous pedicle screw fixation (PPSF). **(C)** Transverse CT imaging three years after OLIF combined with percutaneous pedicle screw fixation (PPSF).

**Figure 2:**
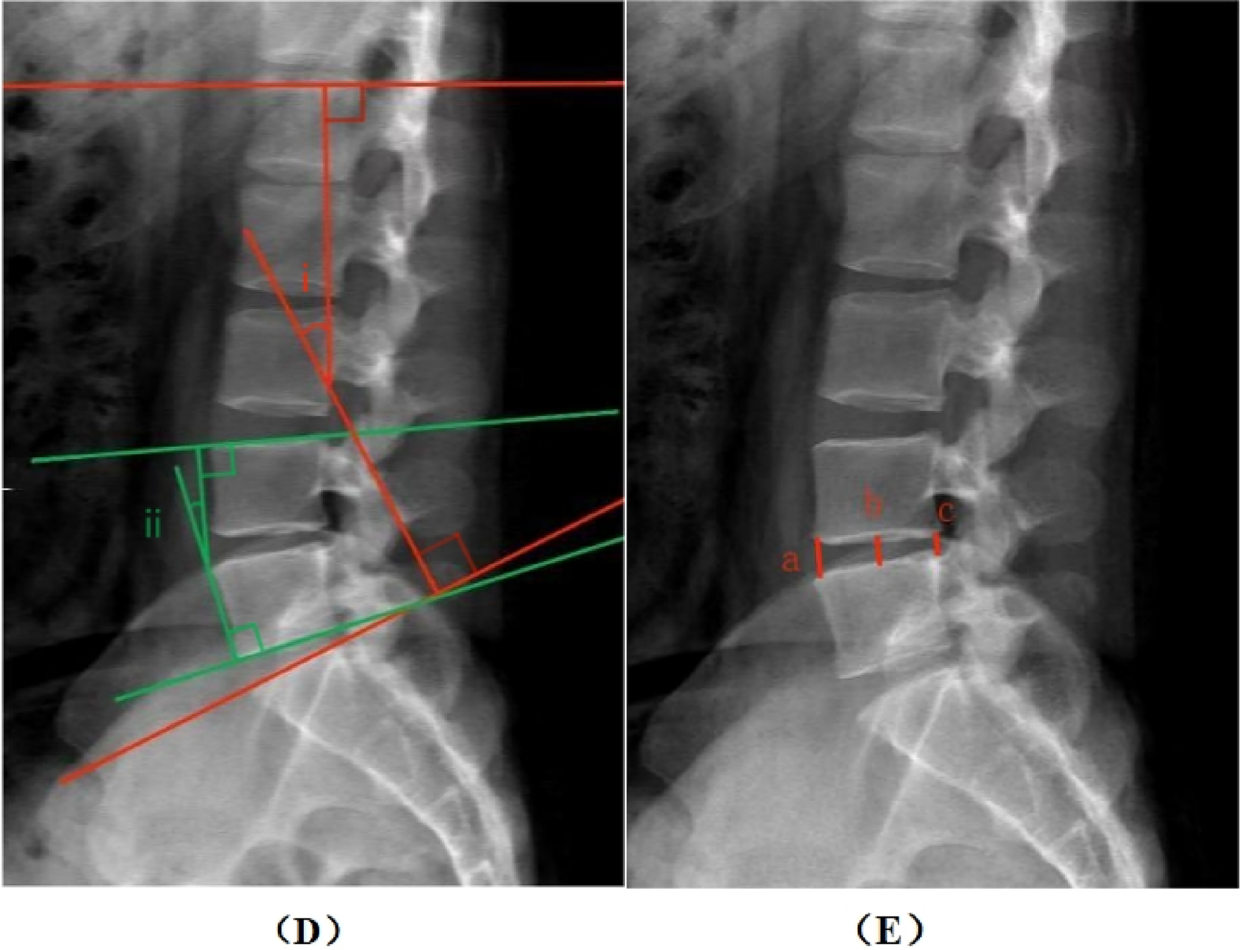
Measurement method. **(D)** the whole lumbar lordotic angle (i) and the segmental lordotic angle (ii). **(E)** Anterior, mid, and posterior margin disc height (a, b, c).

Disc height (DH): The height of the intervertebral disc space was obtained from the average of the anterior, middle, and posterior disc heights, which were repeated three times and averaged (Figures 1, 2) (24).

The efficacy was evaluated by VAS pain score and RMDQ score.

Preoperative low back pain score: RMDQ and VAS were measured before surgery, not at the first visit.

Postoperative low back pain score: RMDQ and VAS were measured at the third-year follow-up. We evaluated the degree of low back pain in the third year after surgery, not only to reduce the neuromuscular pain caused by the operation, but also to observe the influence of different factors on the degree of low back pain relief after surgery.

Patients with RMDQ improvement rate ≥25% and VAS improvement index >3 were considered to have good and effective improvement of low back pain after OLIF.

The improvement rate of RMDQ = (preoperative RMDQ-postoperative RMDQ)/preoperative RMDQ.

VAS improvement index = preoperative VAS- postoperative VAS.

### Statistical analysis

All statistical analyses were performed using SPSS 26.0. Data are presented as mean SD or *n* (%) of patients. Chi-square test, non-parametric test, independent sample T test was used to compare the demographic characteristics and clinical data between groups. Univariate and multivariate unconditional logistic regression were used to estimate crude and adjusted odds ratios and 95% confidence intervals, which are measures of association between risk factors and LBP. For correlation analysis, Pearson's correlation coefficient was used to assess the relationship between variables. All reported *P* values are two-sided, and a *P* value of less than 0.05 was considered to indicate statistical significance.

## Results

This retrospective study recruited 158 patients with low-grade degenerative lumbar spondylolisthesis who underwent OLIF surgery. Of these, 116 completed the final follow-up 36 months after surgery. There were 55 males and 61 females with an average age of 58.61 years (39–73 years). The complete follow-up rate was 73.4% (116 of 158 patients). Thirty-four patients were lost to follow-up. Eight patients who underwent a second operation due to infection, adjacent stage lesions, acute nerve edema, etc. were excluded from the study.

Table 1 shows the demographic and diagnostic characteristics of the patients. At the final follow-up, the preoperative and postoperative lumbar lordosis Angle, segmental lumbar lordosis Angle, intervertebral disc height, VAS score, and RMDQ score were compared, and the differences were statistically significant (*P* < 0.05). After OLIF, the mean ± SD lumbar lordosis Angle changed from 50.00° ± 5.09° to 52.48° ± 5.06°, and the mean ± SD segmental lordosis Angle changed from 13.83° ± 2.60° to 14.82° ± 2.58°. The mean ± SD disc height decreased from 7.53 ± 1.33 mm preoperatively to 10.30 ± 1.50 mm postoperatively. The mean ± SD VAS score decreased from 7.32 ± 0.75 preoperatively to 3.48 ± 1.39 at the final follow-up 3 years postoperatively. The mean ± SD RMDQ score decreased from 14.57 ± 1.91 before surgery to 10.18 ± 2.34 at the final follow-up at 3 years after surgery.

**Table 1:** The demographic and clinical characteristics of patients with mild isthmus were retrospectively studied.

Demographic	Descriptive	Study cohort *n* = 116	*P* value
Age (years)		58.61 ± 7.01	
BMI		23.59 ± 2.44	
Duration of symptoms (months)		27.01 ± 11.92	
Gender	Male	55 (47.4%)	
	Female	61 (52.6%)	
Grade of slippage	1	71 (61.2%)	
	2	45 (38.8%)	
Diabetes	Yes	39 (33.6%)	
	No	77 (66.4%)	
Hypertension	Yes	37 (31.9%)	
	No	79 (68.1%)	
Coronary heart disease	Yes	42 (36.2%)	
	No	74 (63.8%)	
Osteoporosis	Yes	48 (41.4%)	
	No	68 (58.6%)	
Lumbar lordosis Angle (°)	Preoperation	50.00 ± 5.09	0.000
	Postoperation	52.48 ± 5.06	
Segmental lordosis Angle (°)	Preoperation	13.83 ± 2.60	0.004
	Postoperation	14.82 ± 2.58	
Disc height changes (mm)	Preoperation	7.53 ± 1.33	0.000
	Postoperation	10.30 ± 1.50	
VAS	Preoperation	7.32 ± 0.75	0.000
	Postoperation	3.48 ± 1.39	
RMDQ	Preoperation	14.57 ± 1.91	0.000
	Postoperation	10.18 ± 2.34	

Table 2 shows the relationship between demographic characteristics, clinical characteristics and preoperative VAS pain scores as well as preoperative RMDQ scores. Among the statistical characteristics, we did not find any factor that had a significant difference in both VAS score and RMDQ score. However, the preoperative RMDQ scores of low back pain were significantly different between patients with osteoporosis and patients without osteoporosis (*P* < 0.05), and the preoperative RMDQ scores of L4 segment spondylolisthesis and L2/L3 segment spondylolisthesis were significantly different (*P* < 0.05). There was no significant difference between other factors and preoperative VAS score of low back pain and preoperative RMDQ score.

**Table 2:** Univariate analysis evaluated the relationship between demographic characteristics, clinical features, and preoperative visual analogue scale (VAS) pain scores.

Demographic	Descriptive	Number of patients	Pre VAS	*P* value	Pre RMDQ	*P* value
Gender	Male	55	7.38 ± 0.65	0.353	14.44 ± 1.94	0.481
	Female	61	7.26 ± 0.83		14.69 ± 1.89	
Age (years)	≤60	74	7.32 ± 0.74	0.895	14.58 ± 1.94	0.850
	>60	42	7.31 ± 0.78		14.55 ± 1.88	
BMI	≤24.0	74	7.31 ± 0.74	0.667	14.65 ± 2.03	0.603
	>24.0	42	7.33 ± 0.79		14.43 ± 1.70	
Grade of slippage	1	71	7.39 ± 0.71	0.295	14.27 ± 1.99	0.026
	2	45	7.20 ± 0.81		15.04 ± 1.71	
Slippery segments	L4	72	7.35 ± 0.75	0.578	14.85 ± 1.90	0.035
	L2/L3	44	7.27 ± 0.76		14.11 ± 1.87	
Diabetes	Yes	39	7.33 ± 0.74	0.793	14.41 ± 1.68	0.504
	No	77	7.31 ± 0.77		14.65 ± 2.02	
Hypertension	Yes	37	7.35 ± 0.75	0.959	14.43 ± 1.92	0.481
	No	79	7.30 ± 0.76		14.63 ± 1.92	
Coronary heart disease	Yes	42	7.33 ± 0.69	0.922	14.62 ± 0.81	0.839
	No	74	7.31 ± 0.79		14.54 ± 1.98	
Osteoporosis	Yes	48	7.29 ± 0.65	0.837	15.02 ± 1.83	0.033
	No	68	7.34 ± 0.82		14.25 ± 1.92	
Duration of symptoms(months)	≤36	68	7.31 ± 0.83	0.759	14.83 ± 1.74	0.125
	>36	48	7.33 ± 0.63		14.38 ± 2.02	

Table 3 shows the relationships between demographic characteristics, clinical characteristics and postoperative VAS pain scores as well as postoperative RMDQ scores. Preoperative lumbar spondylolisthesis grade, Cage subsidence, duration of preoperative low back pain symptoms more than 36 months, percutaneous pedicle screw implantation, osteoporosis, postoperative segmental lordosis Angle change >0.8°, postoperative intervertebral disc height change >2.5 mm were significantly correlated with postoperative low back pain VAS score and postoperative RMDQ score Significant difference (*P* < 0.05). When the preoperative grade of lumbar spondylolisthesis is greater, preoperative osteoporosis, postoperative Cage subsidence, preoperative low back pain lasting more than 36 months, no percutaneous pedicle screw implantation, postoperative segmental lordosis Angle change ≤0.8°, postoperative intervertebral disc height change ≤2.5 mm, there is no significant difference between the two groups (*P* > 0.05). The postoperative VAS score and RMDQ score of patients with low back pain will be higher, and the postoperative recovery of low back pain symptoms will be poor.

**Table 3:** Univariate analysis evaluated the relationship between demographic characteristics and clinical features, and postoperative visual analogue scale (VAS) pain scores.

Demographic	Descriptive	Number of patients	Post VAS	*P* value	Post RMDQ	*P* value
Gender	Male	55	3.62 ± 1.45	0.310	10.29 ± 2.41	0.585
	Female	61	3.36 ± 1.34		10.08 ± 2.29	
Age (years)	≤60	74	3.69 ± 1.47	0.047	10.22 ± 2.40	0.706
	>60	42	3.12 ± 1.17		10.12 ± 2.25	
BMI	≤24.0	74	3.14 ± 1.16	0.002	10.00 ± 2.42	0.264
	>24.0	42	4.10 ± 1.56		10.50 ± 2.19	
Grade of slippage	1	71	3.17 ± 1.15	0.010	9.45 ± 2.03	0.000
	2	45	3.98 ± 1.60		11.33 ± 2.35	
Slippery segments	L4	72	3.43 ± 1.35	0.634	10.43 ± 2.30	0.194
	L5	44	3.57 ± 1.47		9.77 ± 2.37	
Amount of bleeding	>120 mml	55	3.33 ± 1.25	0.438	9.85 ± 2.35	0.105
	≤120 mml	61	3.62 ± 1.51		10.48 ± 2.31	
Operation duration	>120 min	44	3.45 ± 1.32	0.881	10.23 ± 2.20	0.770
	≤120 min	72	3.50 ± 1.44		10.15 ± 2.44	
Diabetes	Yes	39	3.69 ± 1.36	0.224	10.18 ± 2.34	0.860
	No	77	3.38 ± 1.41		10.17 ± 2.49	
Hypertension	Yes	37	3.70 ± 1.41	0.267	10.27 ± 2.00	0.674
	No	79	3.38 ± 1.38		10.14 ± 2.49	
Coronary heart disease	Yes	42	3.60 ± 1.33	0.439	10.24 ± 2.13	0.844
	No	74	3.42 ± 1.43		10.15 ± 2.46	
Osteoporosis	Yes	48	4.13 ± 1.48	0.000	11.42 ± 2.23	0.000
	No	68	3.03 ± 1.13		9.31 ± 2.01	
Cage subsidence	Yes	47	4.26 ± 1.51	0.000	11.49 ± 2.28	0.000
	No	69	2.96 ± 1.02		9.29 ± 1.93	
Duration of symptoms (months)	≤36	68	3.03 ± 1.12	0.000	9.63 ± 2.18	0.001
	>36	48	4.13 ± 1.50		10.96 ± 2.36	
PPSF	Yes	75	3.16 ± 1.10	0.006		
	9.56 ± 2.05	0.000				
	No	41	4.07 ± 1.66		11.32 ± 2.42	
Lumbar lordosis Angle changes (°)	≤2	40	3.83 ± 1.60	0.128	10.75 ± 2.50	0.037
	>2	76	3.30 ± 1.24		9.88 ± 2.21	
Segmental lordosis Angle changes (°)	≤0.8	35	4.40 ± 1.52	0.000	11.17 ± 2.43	0.002
	>0.8	81	3.09 ± 1.13		9.75 ± 2.18	
Disc height changes (mm)	≤2.5	35	4.26 ± 1.50	0.000	11.57 ± 2.25	0.000
	>2.5	81	3.15 ± 1.21		9.58 ± 2.12	

Patients with RMDQ improvement rate ≥25% and VAS improvement index >3 were considered to have good and effective improvement of low back pain after OLIF, and patients with RMDQ improvement rate <25% or VAS improvement index ≤3 were classified as unsatisfactory improvement of low back pain symptoms after OLIF and poor efficacy group.

Table 4 shows the relationship between demographic characteristics, MRI findings, and efficacy of postoperative LBP improvement. Age >60 years (*P* = 0.015), BMI > 24 kg/m^2^ (*P* = 0.000), larger spondylolisthesis grade (*P* = 0.000), osteoporosis (*P* = 0.000), cage subsidence (*P* = 0.000), duration of symptoms >36 months (*P* = 0.000), no PPSF(*P* = 0.000), lumbar lordosis Angle increase ≤2°(*P* = 0.003), anterior segment Patients with an increase in convex Angle ≤0.8°(*P* = 0.000) and an increase in disc height ≤2.5 mm (*P* = 0.000) had poor postoperative improvement of low back pain. These factors are possible risk factors for the improvement of low back pain after OLIF.

**Table 4:** Univariate analysis assesses the relationship between demographic characteristics, magnetic resonance imaging findings, and improvement in postoperative low back pain.

Demographic	Descriptive	Therapeutic effect		*P* value
		Good 83	Poor 33	
Gender	Male	37	18	0.087
	Female	50	11	
Age(years)	≤60	37	5	0.015
	>60	50	24	
BMI(kg/m^2^)	≤24.0	64	10	0.000
	>24.0	23	19	
Grade of slippage	1	65	6	0.000
	2	22	23	
Slippery segments	L4	30	14	0.194
	L5	57	15	
Amount of bleeding (mml)	>120	45	10	0.134
	≤120	42	19	
Operation duration (min)	>120	34	10	0.825
	≤120	53	19	
Diabetes	Yes	29	10	1.000
	No	58	19	
Hypertension	Yes	27	10	0.819
	No	60	19	
Coronary heart disease	Yes	32	10	1.000
	No	55	19	
Osteoporosis	Yes	24	24	0.000
	No	63	5	
Cage subsidence	Yes	21	26	0.000
	No	66	3	
Duration of symptoms(months)	>36	25	23	0.000
	≤36	62	6	
PPSF	Yes	68	7	0.000
	No	19	22	
Lumbar lordosis Angle changes (°)	≤2	23	17	0.003
	>2	64	12	
Segmental lordosis Angle changes (°)	≤0.8	15	20	0.000
	>0.8	72	9	
Disc height changes (mm)	≤2.5	15	20	0.000
	>2.5	72	9	

The possible risk factors in Table 4 that showed a significant difference in the degree of improvement in efficacy were included in the Logistic regression model. In the multivariate analysis (Table 5), age, BMI, PPSF, change in lumbar lordosis Angle, and change in lumbar segmental lordosis Angle were removed from the logistic regression model, and slippage grade, osteoporosis, cage subsiding, duration of preoperative low back pain symptoms, and improvement in postoperative intervertebral disc height at the surgical level were considered risk factors associated with improvement in postoperative low back pain symptoms. The increase of slip grade (OR = 17.665; 95%CI: 3.262–95.678 *P* = 0.001), disc height increase ≤2.5 mm (OR = 34.377; 95% CI: 5.632–209.818 *P* = 0.000). No osteoporosis (OR = 0.067; 95% CI: 0.013–0.350 *P* = 0.001), no cage subsidence (OR = 0.208; 95% CI: 0.048–0.903 *P* = 0.036), and the duration of preoperative low back pain symptoms ≤36 months (OR = 0.045; 95%CI: 0.007–0.277 *P* = 0.001).

**Table 5:** Logistic regression was used to analyze independent predictors of low back pain relief after OLIF surgery.

Demographic	OR	95%CI		*P* value
		Lower	Upper	
Grade of slippage	17.665	3.262	95.678	0.001
Osteoporosis	0.067	0.013	0.350	0.001
Cage subsidence	0.208	0.048	0.903	0.036
Duration of symptoms	0.045	0.007	0.277	0.001
Disc height changes	34.377	5.632	209.818	0.000

**Figure 3:**
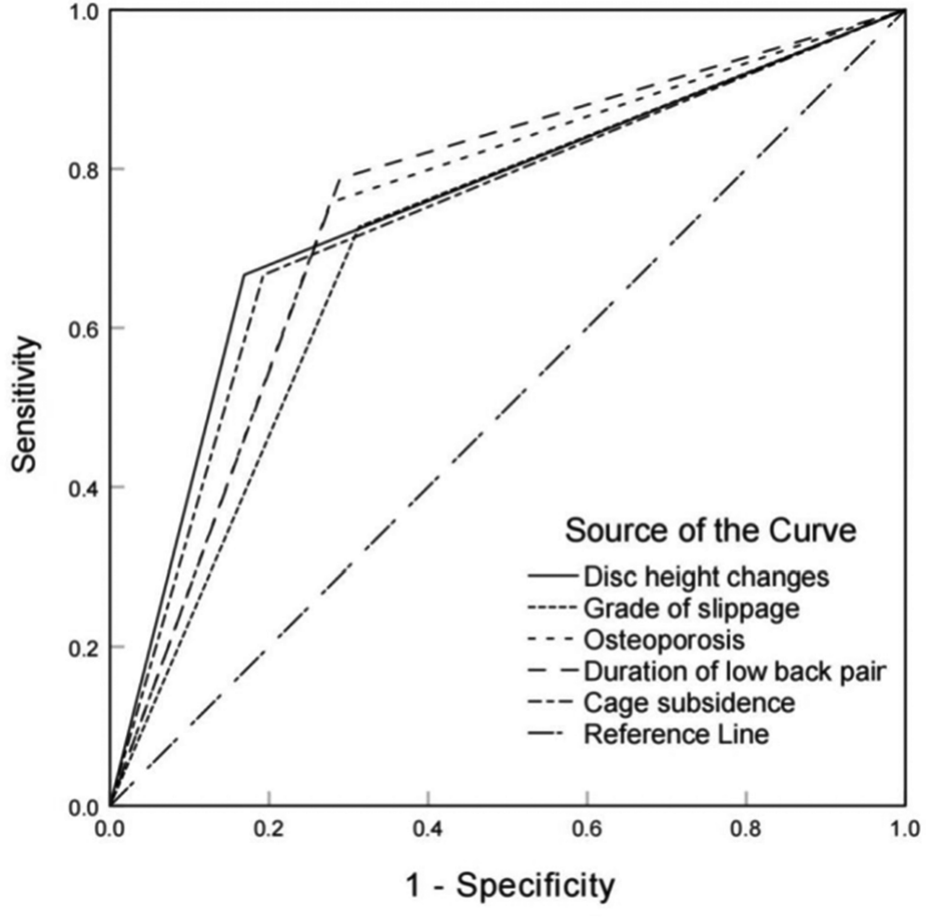
Comparative analysis of the effects of 5 risk factors (grade of slip, osteoporosis, cage subsidence, duration of symptoms of low back pain before surgery, and height of operative intervertebral disc after surgery) on the improvement of the degree of low back pain after OLIF.

We inverted the OR values of risk factors that were negatively associated with improvement of low back pain after OLIF. The receiver operating characteristic (ROC) curves of the 5 risk factors included in the Logistic regression model were drawn by SPSS software to compare and analyze the sensitivity of the 5 risk factors. It can be seen from the figure that the improvement of intervertebral disc height after operation is the most important risk factor for the improvement of low back pain after operation, while the preoperative grade of lumbar spondylolisthesis has the lowest influence on the improvement of low back pain after operation compared with the other four factors (Figure 3).

## Discussion

Degenerative lumbar spondylolisthesis (DLS) is considered as a degenerative disease of the elderly, and its incidence is increasing in today's global society. Low back pain is the main symptom of patients with degenerative lumbar spondylolisthesis (25), and the discomfort caused by it seriously affects the quality of life of the affected patients. Degenerative lumbar spondylolisthesis (DLS) also shows some degenerative imbalance and therefore becomes a risk factor for degenerative scoliosis in later life (26, 27). Lumbar instability is usually the main factor inducing and aggravating low back pain symptoms. OLIF is effective for the treatment of low-grade degenerative lumbar spondylolisthesis. During surgery, cages are used to enlarge degenerative segments and stretch soft tissues such as the ligamentum yelum within the spinal canal, thereby enlarging the volume of the spinal canal and foramina to relieve low back pain (28–31). OLIF allows for more complete disc removal, better indirect decompression, greater bone graft area, and allows for larger cage placement in the epiphyseal ring. This is more conducive to postoperative intervertebral fusion, and these advantages reduce the incidence of cage subsidence, thereby alleviating postoperative LBP (32). Effective fusion of unstable segments has been shown to provide effective relief of low back pain symptoms (33). In addition, this surgical approach does not require dissection of paravertebral tissue, and may not involve removal of bone structures near the nerve root canal, such as articular processes. This procedure has less nerve stimulation and avoids the complications of postoperative lower limb pain and numbness caused by intraoperative nerve stimulation.

In our study of 116 patients who underwent OLIF for low-grade degenerative lumbar spondylolisthesis, There were 33 patients who did not achieve satisfactory improvement of low back pain after operation. The factors related to poor improvement of low back pain symptoms after operation included high grade of spondylolisthesis, osteoporosis, cage subsidence, long duration of preoperative low back pain symptoms (>36 months), and small increase of intervertebral disc height (≤2.5 mm). The small increase of intervertebral disc height (≤2.5 mm) at the surgical level had the greatest impact on the poor improvement of low back pain symptoms. Studies have found that the loss of lumbar curvature and the change of intervertebral disc height (DH) are closely related to the occurrence and development of degenerative lumbar spondylolisthesis (DLS). In addition, other current studies have shown that achieving better disc height (DH) is associated with improvement in postoperative low back pain (LBP), suggesting that regardless of the type of fusion used, surgery should aim to restore disc height to reduce postoperative LBP (34). Sato et al. reported significant improvements in DH and spinal canal area after OLIF surgery. Low back pain and leg pain were significantly reduced (35). This is also consistent with our findings that postoperative disc height recovery is essential for the relief of postoperative low back pain symptoms. It is not only beneficial to restore the patient's original lumbar curvature, but also allows the patient to obtain a better biological force line. It can also effectively expand the height of the intervertebral foramen and relieve the neurological symptoms. In addition, it has been proved that the reconstruction of LL or lumbar segmental lordosis is essential for the recovery of symptoms and the prevention of adjacent segment degeneration even in short-segment surgery (36). However, in our study, the changes of lumbar lordosis Angle and segmental lordosis Angle after operation were significantly different from those before operation. The changes of LL and SL after surgery also make a significant difference in the low back pain scores of patients before and after surgery. In addition, patients with larger and more reasonable changes in LL and SL angles had better relief of low back pain symptoms after surgery, but this factor was not included in our Logistic model and did not become our risk factor. We could then further investigate the relationship between postoperative changes in spine biomechanical Angle and the improvement of outcome in patients with degenerative lumbar spondylolisthesis with low back pain.

After patients undergo OLIF surgery, the stability of the surgical segment is an important rehabilitation indicator of great concern to clinicians (37). In clinical practice, osteoporotic patients with degenerative lumbar diseases usually require OLIF to increase lumbar stability and reduce the risk of fracture and failure. Patients with osteoporosis are often accompanied by the risk of complications such as cage subsidence, internal fixation loosening, and poor interbody fusion after surgery, which lead to low back pain, lower extremity neurological symptoms, and other problems. The effect of surgery is greatly reduced. In addition, due to the low Young's modulus of osteoporotic vertebrae, the difference in mechanical properties between bone and cage leads to increased stress on bone and decreased stress on cage, which indirectly leads to increased risk of cage subsidence. Therefore, the selected fixation system and vertebral strength should be considered when OLIF is performed in osteoporotic patients (38). Studies have shown that patients with dual-energy x-ray absorptiometry (DEXA) T scores <−1.0 undergoing OLIF surgery alone have a higher risk of cage subsidence (39). This has prompted spine surgeons to investigate various methods to alleviate osteoporosis and its various secondary problems. Such as bisphosphonates and recombinant parathyroid hormone to maximize bone quality and surgical outcomes in this patient population (40, 41). Tu et al. (41) showed that intravenous infusion of zoledronic acid, a bisphosphonate, in patients with lumbar disc herniation after lumbar interbody fusion improved joint fusion rates and clinical outcomes, while reducing the risk of compression fracture, screw loosening, and graft subsidence.

In this study, there was no significant difference in the preoperative VAS score and RMDQ score between patients with preoperative low back pain duration >36 months and those with preoperative low back pain duration ≤36 months. However, when evaluating the degree of low back pain after OLIF, it was found that the VAS score and RMDQ score of the patients with the duration of preoperative low back pain symptoms ≤36 months were significantly lower than those of the patients with the duration of preoperative low back pain symptoms >36 months after OLIF, and the difference was statistically significant, and the improvement effect of low back pain was better. At present, there are findings suggesting that patients with low back pain symptoms for 3 years have more severe preoperative LBP. Despite their more severe preoperative LBP, they still achieved satisfactory improvement in postoperative LBP. In our study, although there was no significant difference in the preoperative VAS score and RMDQ score between patients with preoperative low back pain duration >36 months and those with preoperative low back pain duration ≤36 months, the improvement of low back pain after OLIF was significant regardless of the duration of preoperative low back pain. This part is supported by previous studies by this research group (42).

Cage subsidence after OLIF is very common, with a subsidence rate of about 30%, which may affect orthopedic surgery and even lead to decompression failure (43, 44). Cage subsidence may hinder the effect of indirect decompression through disc height reduction and lead to discomfort, nonfusion, and other negative effects. Intraoperative endplate injury was considered to be a disruption (discontinuity or wear) of the endplates of any one or both adjacent vertebral bodies. Severe endplate injury may occur, followed immediately by intraoperative cage subsidence, cage retropulsion or even vertebral fracture (45, 46). OLIF requires a large interbody fusion cage for indirect decompression. When a larger cage is inserted, the stress between the cage and the endplate surface is more prominent, which is more likely to cause endplate injury during operation. In OLIF, the problem of loss of intervertebral height due to fusion subsidence cannot be avoided. Studies have shown that OLIF + PPSF can improve the axial bearing capacity of the fused segment, thereby reducing the incidence of subsidence (47).

In Table 4, it can be seen that PPSF is a statistically significant risk factor for the degree of improvement in efficacy. However, it was excluded from the logistic regression model. The application of PPSF mainly improves the symptoms of low back pain (LBP) by increasing the posterior internal fixation, reducing the subsidence of interbody fusion cage, and improving the early fusion rate. For patients with large bone mass, low body weight, and no intraoperative endplate injury, OLIF alone can better restore the patient's biomechanics, lumbar Angle, height, and relatively solid fixation strength, and can provide better surgical results for patients with degenerative lumbar spondylolisthesis. In addition, it can avoid the damage to the back muscles and reduce the symptoms of low back pain caused by muscle damage and other surgical factors in the early postoperative period. It has been reported that both OLIF alone and OLIF combined with PPSF are safe and effective in the treatment of low back pain, especially in patients with low-grade degenerative lumbar spondylolisthesis. For patients with osteoporosis, intraoperative endplate injury, isthmic spondylolisthesis, obesity, and high activity demand, OLIF combined with PPSF is superior to OLIF alone. PPSF is undoubtedly effective in improving spinal stability and reducing the incidence of cage subsidence. For the choice of PPSF, responsible doctors often need to make a comprehensive assessment of patients before making an appropriate decision (13, 39, 48, 49).

The present study has several limitations: (1) The sample size included was small. (2) This study is a retrospective case-control study. Inevitably, there is a certain degree of selection bias that may affect the results. (3) The PPSF group samples were not included in the logistic regression model, which may be due to the small number of patients undergoing PPSF and insufficient sample size, or it may be due to the correlation between this group and the osteoporosis group and the cage subsiding group, which may affect the model.

## Conclusions

The increase of spondylolisthesis grade, osteoporosis, cage subsidence, the duration of preoperative low back pain symptoms >36 months, and the increase of intervertebral disc height ≤2.5 mm are the influencing factors for the improvement of low back pain symptoms after OLIF for mild degenerative lumbar spondylolisthesis. Among them, the increase of intervertebral disc height ≤2.5 mm is the biggest factor affecting the relief of low back pain symptoms after OLIF. Our findings may help surgeons to identify patients at high risk of poor low back pain outcome after OLIF in patients with mild degenerative lumbar spondylolisthesis, so that they can carry out preoperative intervention in advance of some factors, select appropriate surgical methods, and postoperative rehabilitation methods to reduce or prevent the occurrence of poor low back pain outcome after OLIF, so as to improve surgical outcomes. The purpose of improving patient satisfaction.

## Data Availability

The raw data supporting the conclusions of this article will be made available by the authors, without undue reservation.
